# Cancer-associated fibroblasts: heterogeneity and their role in the tumor immune response

**DOI:** 10.1007/s10238-024-01375-3

**Published:** 2024-06-12

**Authors:** Yuxuan Xiao, Ziyu Wang, Meng Gu, Panjian Wei, Xiaojue Wang, Weiying Li

**Affiliations:** Cancer Research Center, Beijing Chest Hospital, Capital Medical University, Beijing Tuberculosis and Thoracic Tumor Research Institute, Beijing, China

**Keywords:** Cancer-associated fibroblasts, Cancer cells, Heterogeneity, Immunosuppression

## Abstract

In recent decades, many reports have been published on the composition and function of the tumor microenvironment (TME), among which cancer-associated fibroblasts (CAFs) have received much attention. CAFs have different degrees of heterogeneity in terms of their origin, phenotype, and function and can be divided into different subpopulations. These subgroups may play different roles in the occurrence and development of tumors. In addition, CAFs are closely associated with tumor immunity and have been found to regulate immune cell activity and to suppress the tumor immune response. In this review, we systematize the heterogeneity and characteristics of CAFs, discuss how specific CAF subgroups contribute to cancer progression by inducing an immunosuppressive microenvironment, and finally, we examine the future clinical applications of CAF subgroups.

## Introduction

The TME is a complex and unique system composed of tumor cells, CAFs, immune cells, mesenchymal tissue, blood vessels, various growth factors, cytokines and chemokines. Together, these different cellular and acellular components drive tumor growth, invasion, metastasis, and response to therapies. CAFs are the most abundant group of stromal cells in the tumor microenvironment. Increasing evidence suggests that CAFs play a critical role in tumor proliferation, metastasis, and invasion by interacting with tumor cells in multiple ways to promote tumor growth and a maintain long-term propensity toward malignancy [1–3]. In addition, CAFs can secrete a variety of cytokines and growth factors involved in tumor drug resistance, epithelial–mesenchymal transition (EMT), endothelial-to-mesenchymal transition (EndMT), tumor remodeling, metabolism, and tumor regeneration [4–7].

Initially, CAFs were believed to be a homogeneous class of cells, but as research progressed, researchers discovered that CAFs are highly heterogeneous, as reflected by differences in their origin, function, phenotype and biomarkers [1, 8, 9]. Although most studies have demonstrated that CAFs can promote tumor proliferation, some researchers have found that a subset of CAF subgroups exert tumor-suppressive effects [10, 11]. In addition, some CAF subgroups can participate in the signaling mechanisms by which tumor cells evade immune surveillance, mediate tumor immune tolerance, and even influence the therapeutic effect of anticancer drugs [12, 13]. Some CAF subsets can also increase the number of regulatory T lymphocytes and suppress the activity of effector and cytotoxic immune cells. These functions are mainly implemented through the secretion of cytokines, chemokines and growth factors [14, 15]. With further research, additional subtypes of CAFs have been identified, which has increased our understanding of CAFs. If we can fully understand the function of CAF subsets, we can inhibit tumor proliferation through targeted therapy. Therefore, to present a detailed analysis of CAFs, we reviewed these vastly different subgroups in terms of their origins, functions, phenotypes, immunosuppression capacity, and other aspects.

## Heterogeneity in the origin of CAFs

Various lines of evidence suggest that CAFs have many different origins [16]. Initially, most researchers supported the idea that CAFs originate from normal fibroblasts (NFs). These NFs are distributed around tumors and can be activated to form CAFs after specific signaling pathways, including the EMT and SMAD pathways, are triggered by transforming growth factor-β (TGF-β) and platelet-derived growth factor (PDGF) signaling. In addition, the P38/MAPK pathway leads to enhanced cell-to-cell adhesion [17–20]. Fibroblast precursor cells in the TME can also directly transform into CAFs through mesenchymal transition [21]. Notably, a strong correlation between the expression level of NADPH oxidase subunit 4 (NOX4) and the number of CAFs in tumor tissues has been observed in a variety of tumor cells, which suggests that the conversion of NFs to CAFs is dependent on the NOX4 enzyme [22, 23]. In addition, CAFs can originate from unique tissue-resident cells, such as pancreatic stellate cells and hepatic stellate cells [24, 25].

Bone marrow-derived mesenchymal stem cells (BM-MSCs) are another important source of CAFs. Although the quantity of these cells is small, cells with multidirectional differentiation potential have previously been shown to differentiate into CAFs through mesenchymal transition. According to several reports, BM-MSCs can differentiate into CAFs by upregulating Calponin 1, α-SMA and collagen through the MRTF transcription factor [26, 27]. By continuous stimulation with TNF-α+IL-1β, BM-MSCs can convert into inflammatory CAFs that promote tumor development. Some evidence also indicates that the process by which BMSCs differentiate into CAFs can be upregulated by Clusterin to enhance tumor angiogenesis [28, 29]. In addition, Smad3 has been reported to promote CAF generation via macrophage-myofibroblast transition [30].

**Fig. 1:**
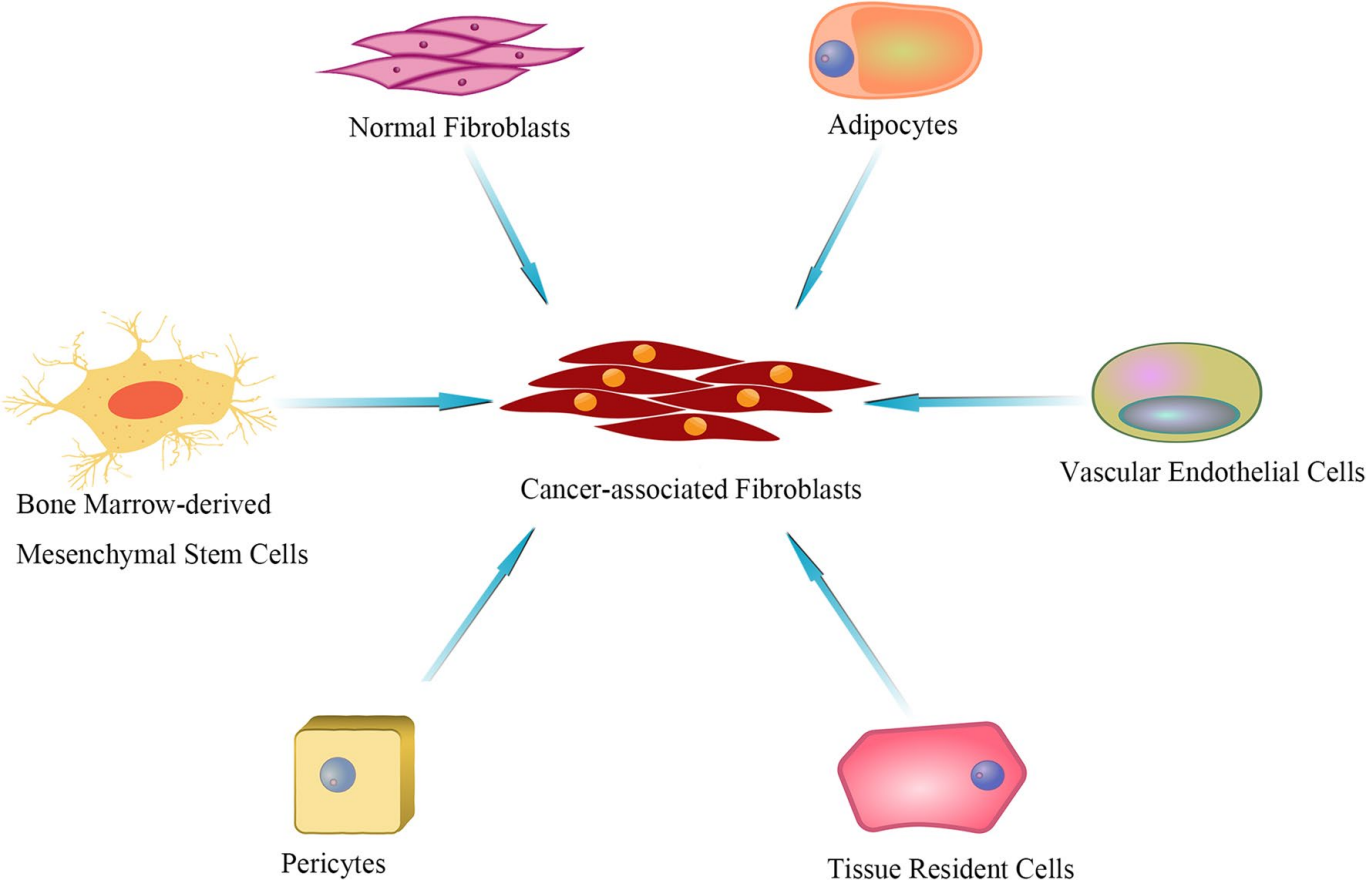
Several origins of CAF. In tumors, normal fibroblasts, adipocytes, vascular endothelial cells, tissue-resident cells, pericytes and bone marrow-derived mesenchymal stem cells can be transformed into CAF, showing the heterogeneity of CAF origin

In addition to the generally recognized sources of CAFs mentioned above, some studies have shown that CAFs can develop from other cell types. Adipocytes can also become adipocyte-derived fibroblasts (ADFs) through phenotypic changes. It was first reported in breast cancer that adipocytes exhibit increased secretion of fibrin and collagen I via a mechanism in which tumor cells activate the Wnt/β-catenin pathway to secrete Wnt3a, which leads to the dedifferentiation of adipocytes [31]. Human platelet-derived growth factor-BB (PDGF-BB) induces the transformation of pericytes into CAFs via the induction of mesenchymal transdifferentiation of human microvascular endothelial cells into CAFs through TGF-β2 signaling [32]. Regardless of the origin of CAFs, these cells change to adapt to the tumor microenvironment (Fig. 1).

## Heterogeneity of CAF biomarkers

Due to the heterogeneity of CAFs, numerous biomarkers have been discovered, and to date, dozens of markers have been shown to define CAFs or a CAF subgroup. Some of the more familiar markers, such as fibroblast activation protein (FAP), platelet-derived growth factor receptor-alpha/beta (PDGFR-α/β), ferroptosis suppressor protein 1 (FSP1), vimentin, fibronectin, and alpha-smooth muscle actin (α-SMA), as well as some newly defined “niche” markers, such as CD90, tenascin-C, periostin, desmin, Thy-1 (THY1), podoplanin (PDPN), integrin subunit beta 1 (ITGB1), and caveolin 1 (CAV1), have been identified in recent years [9, 33–39]. Studies using α-SMA to define CAFs in human tumors have shown that they accumulate in cancers with a poor prognosis, especially in breast cancer [40–42]. High expression of PDGFR-β was associated with a poor prognosis in in situ ductal carcinoma [43, 44]. The value of CAV1 or FSP1 in predicting the prognosis of patients with breast cancer based on CAFs has been confirmed, although some of the results are conflicting [45–47]. In addition, FAP is abundant in the stroma of aggressive breast cancer [48–51]. Two discrete populations of FAP^+^ mesenchymal cells can also be distinguished according to PDPN expression. Although FAP^+^PDPN^+^ CAFs and FAP^+^PDPN^−^ CAFs have been shown to express high levels of ECM components, FAP^+^PDPN^+^ CAFs were enriched according to the transcriptomic data and were associated with TGF-β signaling. Moreover, this CAF subset is located mainly on the outer edge of the tumor and in close contact with T-cells, whereas FAP^+^PDPN^−^ CAFs are located mainly around vessels [49]. Although biomarkers of CAFs are continuously being discovered, none of these markers is highly specific. For example, some normal stromal cells can also express vimentin, fibronectin and FAP. α-SMA, which is commonly used in studies on CAFs, is also commonly expressed in blood vessel walls and the intestinal muscularis mucosa. On the one hand, the lack of specific markers is one of the greatest challenges in CAF research, as this increases the difficulty in locating the desired group of cells. On the other hand, the lack of markers also helps to pinpoint the cancer-promoting and cancer-suppressing subpopulations of CAFs, thus showing their high potential value as prognostic factors and therapeutic targets.

Research on CAFs in PDAC has revealed three CAF subsets. In the TME of PDAC, two different CAF subtypes characterized by either a myofibroblastic or an inflammatory phenotype through transforming growth factor (TGF-β) and IL-1/JAK-STAT signaling as the major pathways have been identified [52]. As early as 2018, a team used the cell surface markers CD10 and GPR77 to define a group of CAFs in non-small cell lung cancer and reported that this subpopulation maintains tumor cell stemness and promotes chemoresistance; they also reported that targeting this subpopulation restores sensitivity to chemotherapeutic agents [53]. In addition, some specific CAF subgroups have been identified in recent years: inflammatory CAFs (iCAFs) that express inflammatory markers, such as interleukin 6 (IL-6) and leukemia inhibitory factor (LIF) [54], myofibroblast CAFs (myCAFs) that express myofibroblast markers, such as α-SMA [52], antigen-presenting CAFs (apCAFs) that express MHC-II and CD74 [55], and secretory CAFs that develop hypofibrotic characteristics following alterations in CAFs induced by Kras and CXCR2 signaling [56].

## Functional heterogeneity of CAFs

These different sources of CAFs are numerically dominant and serve as the most dominant stromal cell population in the TME, but their functions are not homogeneous. CAFs can be divided into different subgroups according to their varied functions in many types of cancers. Four subpopulations of CAFs are present in the axillary lymph nodes of metastatic breast cancer, CAF-S1 to CAF-S4, of which the CAF-S1 and CAF-S4 subpopulations are the most numerous and are positively correlated with tumor cell invasion. CAF-S1 recruits CD4+CD25+T lymphocytes to generate an immunosuppressive environment in a manner dependent on the chemokine CXC ligand 12 (CXCL12). CAF-S4 enhances the contractility of other CAFs to enhance the motility and invasiveness of cancer cells through the NOTCH signaling pathway [51]. Four subpopulations of CAFs were identified in a mouse model of breast cancer: vascular CAFs (vCAFs), matrix CAFs (mCAFs), cycling CAFs (cCAFs) and developmental CAFs (dCAFs). vCAFs originate from the perivascular area, mCAFs are the progeny of normal fibroblasts, cCAFs represent the proliferative state of vCAFs, and dCAFs originate from tumor cells and undergo EMT [57]. According to another study on non-small cell lung cancer, CAFs may be classified into three subgroups based on their characteristics: class I CAFs cause drug resistance mainly through the expression of hepatocyte growth factor (HGF) and recombinant fibroblast growth factor 7 (FGF7), while the administration of MET and FGFR inhibitors can reduce resistance; class II CAFs lead to drug resistance through high FGF7 expression, while the administration of fibroblast growth factor receptor (FGFR) inhibitors can improve the efficacy of targeted drugs; class III CAFs do not show a weak correlation with the response to targeted therapies. However, class III CAFs can enhance the infiltration of immune cells and are correlated with the number of CD8^+^ T-cells in tumor lesions, which may be useful for subsequent immunotherapeutic strategies [58]. Two subgroups of CAFs, CAF-A and CAF-B, have also been identified in colorectal cancer. CAF-B cells express genes associated with extracellular matrix remodeling, whereas CAF-A cells express cytoskeletal genes and other markers that activate myofibroblasts, including matrix metalloproteinase 2 (MMP2), a TGF-β activator [59]. Researchers have also found that CD105 expression is a marker of two stable and functionally distinct pancreatic fibroblast cell lines: CD105-positive CAFs favor tumor growth in vivo, while CD105-negative CAFs exhibit high tumor-suppressive behavior [11]. A subpopulation of CAFs that express CD16 has also been identified in breast cancer, where CD16^+^ fibroblasts reduce drug delivery by enhancing extracellular matrix stiffness in response to trastuzumab stimulation. The interaction between trastuzumab and CD16 activates the intracellular SYK-VAV2-RhoA-ROCK-MLC2-MRTF-A pathway, which leads to increased contractility and matrix production [60]. Notably, SLC14A1+CAFs are induced by interferon signaling and confer stemness to bladder cancer cells via the WNT5A paracrine pathway [61].

Although most studies support the finding that CAFs can promote tumor growth, limited evidence demonstrates that CAFs can also inhibit tumor growth [11]. For example, in one study, the ability of pancreatic ductal adenocarcinoma (PDAC) to grow was enhanced in the presence of fewer CAFs, while in a genetically engineered mouse model of PDAC, removal of CAFs resulted in decreased tumor angiogenesis and longer survival, which suggests that CAFs driven by hedgehog factors can inhibit tumor development [62]. Other studies have shown that an absence of CAFs leads to the development of aggressive tumors and reduced survival in vivo, as the number of cancer stem cells (CSCs) increases [39, 63]. Recently, CD146+CAFs, CAV1high CAFs and PDGFRα^+^Saa3^−^ CAFs have been identified as tumor-suppressive CAF subgroups in breast cancer [64, 65]. These studies suggest that certain CAF subgroups can prevent cancer growth, but biomarkers of these CAFs need to be further investigated to determine which subgroups are specifically involved in cancer growth inhibition. In recent years, interest has increased in the relationship between CAFs and tumor drug resistance. CAFs can affect the metabolism of antitumor drugs, impede drug delivery, and participate in the mechanisms of chemotherapy resistance [4]. In gastric cancer, cisplatin and paclitaxel promote the secretion of miR-522 by CAFs through activation of the USP7/hnRNPA1 axis, which leads to ALOX15 inhibition and a reduction in lipid ROS accumulation in cancer cells, ultimately leading to a decrease in sensitivity to cancer chemotherapy [66]. In non-small cell lung cancer, CAFs that express the zinc transporter protein ZIP1 promote chemoresistance by Zn2^+^ transfer and by upregulating connexin 43 protein (CX43) to increase their adherence to cancer cells [67]. Moreover, in EGFR-mutant lung cancers, CAF paracrine secretion of the pro-oncogenic cytokine IGF is reduced, and the level of the anti-oncogenic protein IGFBP is increased, which attenuates the activation of IGF1R and integrins and inhibits IGF1R-mediated IRS2/AKT signaling and integrin-mediated FAK/ERK signaling, thereby enhancing the efficacy of EGFR inhibitors [68]. Three previously mentioned functionally defined CAF isoforms can be used in different therapeutic strategies to overcome CAF-mediated therapeutic resistance, which makes CAFs a popular target in studies of tumor-targeted therapies [58]. In addition, in a PDAC mouse model, losartan was shown to enhance the efficacy of chemotherapeutic agents by modulating CAFs and the extracellular matrix and by improving tumor vascular perfusion and hypoxia, which indicates that targeting CAFs are a promising and realistic therapeutic strategy [69] (Table 1).

**Table 1:** Heterogeneity of CAF subgroups

Subsets	Markers	Signaling Pathways	Functions	Organ	Ref
Inflammatory fibroblasts	PDPN, lL-6, α-SMA^Low^, LIF	NF-kB signaling	Melaslasis, Angiogenesis, Immunosuppression	Pancreas	[54]
Myofibroblasts	α-SMA^High^, FAP^+^, CTGF^+^, TNC^+^,TAGLN^+^	(TGF)- β and lL-l/JAK-STAF signaling	Migration, Invasion, Metastasis	Pancreas	[52]
Antigen-presenting CAFs	PDPN, Saa3, MHC-IIgene, CA74	MTORC1 signaling	Immunosuppression	Pancreas	[55]
Secretory CAFs	FAP, α-SAM, CD10	Kras-CXCR2 signaling	Proliferation, Angiogenesis	Pancreas	[56]
CAF-S1	FAP^High^, CD29^Med-high^, α-SMA^High^, PDPN^High^, PDGFRβ^High^	TGF-β signaling, CXCL12 signaling	Proliferation, Migration, Invasion, Metastasis	Breast	[51]
CAF-S2	FAP^Neg^, CD29^Low^, α-SMA^Neg-Low^, PDPN^Low^, PDGFRβ^Low^	Not describe	Not describe	Breast	[51]
CAP-S3	FAP^Neg-Low^, CD29^Med^, α-SMA^Neg-Low^, PDPN^Low^, PDGFRβ^Low-Med^	Not describe	Not describe	Breast	[51]
CAF-S4	FAP^Low-Med^, CD29^High^, α-SMA^High^, PDPN^Low^, PDGFRβ^Med^	Not describe	Proliferation, Migration, Invasion, Metastasis	Breast	[51]
vCAFs	PDGFRα, Nidogen-2, Desmin, CD31	PDGF-CC signaling	Angiogenesis	Breast	[57]
mCAFs	PDGFRα, Fibulin-l	PDGF-CC signaling	Immunosuppression	Breast	[57]
cCAFs	Not describe	PDGF-CC signaling	Not describe	Breast	[57]
dCAFs	PDGFRα, SCRGl	PDGF-CC signaling	Migration	Breast	[57]
CD10^+^/GPR77^+^CAF	CD10, GPR77, IL-6 α-SMA, PDGFRβ, FAP, FSP1, Collagen1	NF-kB signaling	Proliferation, Migration, Chemoresistance	Breast	[58]
CAF-A	MMP2, DCN, COL1A2	Not describe	Not describe	Colorectal	[59]
CAF-B	ACTA2, TAGLN, PDGFA	Not describe	Not describe	Colorectal	[59]
CD105^Pos^CAF	CD105^pos^, FAP, Ly6cl	TGF-β signaling	Proliferation	Pancreas	[11]
CD105^Neg^CAF	CD105^Neg^, ITG26, CD74, MHC-II	Not detailed	Inhabitation	Pancreas	[11]
CD16^+^CAF	CD16^+^, αSMA^+^, FAP, CD10^-^, GPR77^-^	SYK-VAV2-RhoA-ROCK-MLC2-MRTF-A pathway	Drug Resistance	Breast	[60]
SLCWA1^+^irCAF	SLC14A1	WNT5A/β-catenin pathway	Promote stemness	Bladder	[61]

## Role of CAFs in the antitumor immune response

The heterogeneity of various types of CAFs is well established, and likewise, the role of CAFs in the regulation of antitumor immune responses is not negligible. The tumor microenvironment contains numerous immunosuppressive cells, such as Tregs, tumor-associated macrophages (TAMs) and myeloid-derived suppressor cells (MDSCs), which amplify and activate specific signaling pathways in the bodies of cancer patients. In addition, a number of cytokines and chemokines, such as TGF-β, vascular endothelial growth factor-A (VEGFA), prostaglandin E_2_ (PGE_2_), and indoleamine 2,3-dioxygenase (IDO), can also modulate the immune response. CAFs can affect the immune response within the tumor via association with these immunosuppressive cells or by cytokine secretion. Some CAFs not only promote tumor development, but they also regulate the extracellular matrix and create a barrier to drug and immune cell infiltration. Furthermore, CAFs can also affect metabolism by producing high levels of lactic acid generated by glycolysis, thus forming an acidic microenvironment, which in turn inhibits immune cell activity [70].

Among immune cells, T lymphocytes (T cells) are undoubtedly the main force involved in tumor killing, and they play an important role in the regulation of adaptive immune responses. However, CAFs have been shown to cross-present exogenous antigens and induce T-cell death through PD-L2 and FASL [71]. They can also inhibit T-cell motility and infiltration, which promotes tumor immunosuppression [72]. In non-small cell lung cancer, researchers have identified two subtypes of CAFs associated with T-cell exclusion, MYH11+αSMA+CAFs and FAP+αSMA+CAFs, both of which are capable of depositing around the tumor parenchyma and promoting T-cell rejection by producing collagen fibers [73]. CAFs also secrete a variety of cytokines that directly affect T-cell function. For example, CAFs with high FAP expression alone promote immunosuppression of the TME in colorectal cancer by upregulating CCL2 secretion, increasing myeloid cell retention, and reducing T-cell activity [74]. Furthermore, CAF-mediated immunotherapy resistance can be effectively overcome through NOX4 inhibition, and pharmacologic inhibition of NOX4 potentiates immunotherapy by overcoming CAF-mediated CD8+T-cell exclusion [75]. Dendritic cells are able to phagocytose, process and present antigens to initiate T-cell-mediated immune responses, and their expression of high levels of class I and II MHC complexes, adhesion molecules and costimulatory molecules can lead to the activation of T-cell functions, whereas CAFs can prevent T-cell activation by DCs through direct or indirect pathways [76]. IDO and vascular endothelial growth factor produced by CAFs can inhibit the activity of DCs and affect their antigen-presentation function [77]. In addition, CAFs enable DCs to inhibit T-cell proliferation by inducing CD4+CD25+Foxp3+Tregs and reducing IFN-γ production by CTLs [77]. Natural killer (NK) cells are derived from bone marrow hematopoietic stem cells and have both cytotoxic and immunomodulatory functions. CAFs impair the antitumor capacity of NK cells by inducing ferroptosis, which conversely, also decreases intracellular iron levels to protect NK cells against CAF-induced ferroptosis [78]. In addition, TGF-β secreted by CAFs in pancreatic tumors can reduce the expression of NKG2D receptors on NK cells [79]. Another study showed that CAFs can inhibit the killing activity of NK cells and promote cancer development by decreasing the expression of the poliovirus receptor (PVR) on the cell surface [80].

## CAFs and immunosuppressive cells

**Fig. 2:**
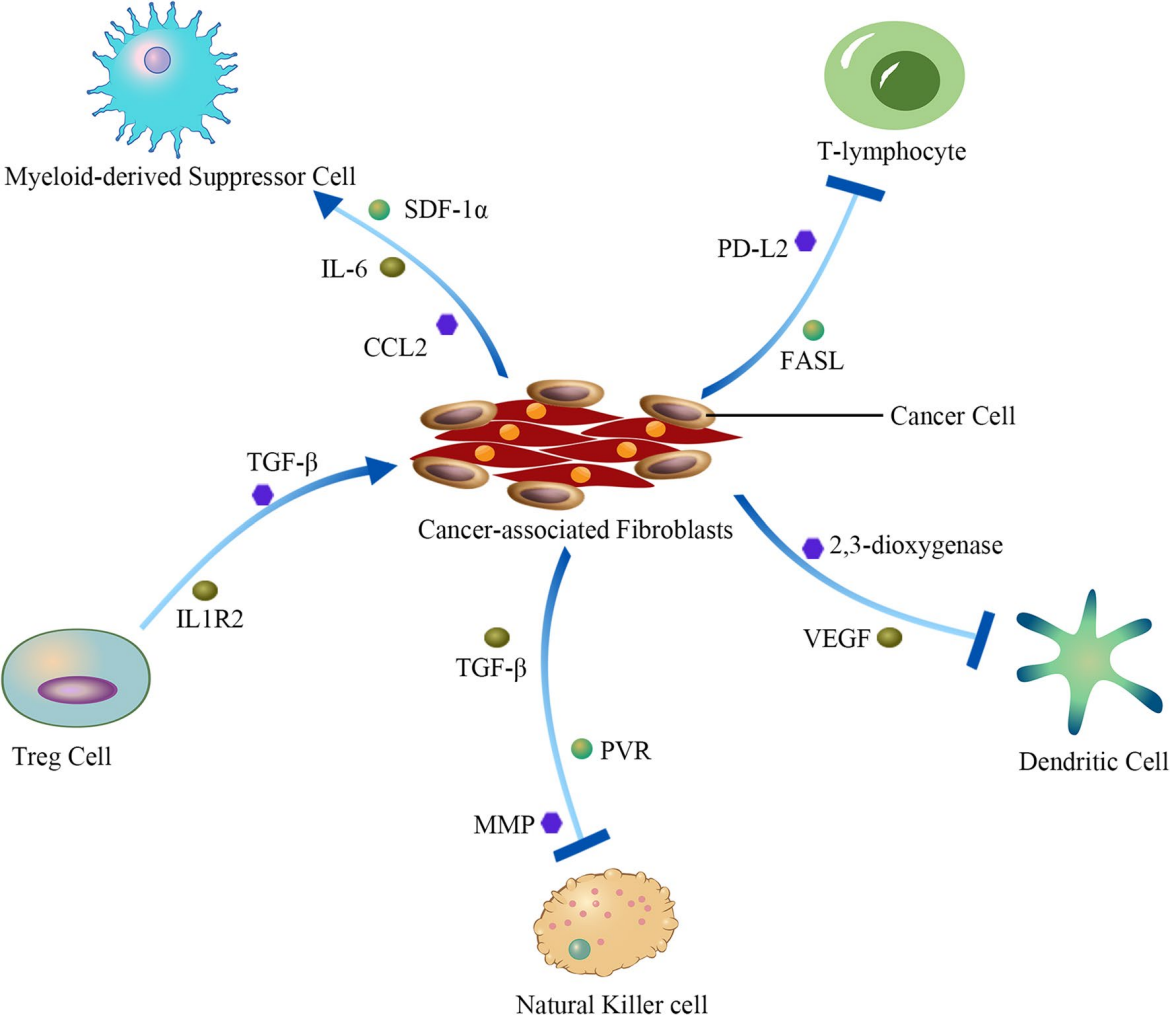
Interaction between CAF and immune cells. CAF induces T-cell death through PD-L2 and FASL; IDO and VEGF secreted by CAF can inhibit the activity of DC; TGF-β secreted by CAF can reduce the expression of NKG2D, NKp30 and NKp44 receptors on NK cell. CAF inhibit the activity of NK cell by reducing PVR; TGF-β secreted by Tregs induces CD8+T cell apoptosis by promoting the conversion of NF to CAF. IL1R2+Tregs up regulate MHC-II expression in CAF; CAF secretes IL-6 and SDF-1α to induce the activation of MDSC. CAF with high FAP expression up regulates the secretion of CCL2 through STAT3 signal to recruit MDSC

Many immunosuppressive cells are also present in the TME where they exert negative immunomodulatory effects. These cells significantly inhibit T-cell infiltration and function, which leads to tumor invasion and metastasis, and can also cause tumors that initially respond to immune checkpoint inhibitors to become resistant to treatment at a later stage. Regulatory T-cells (Tregs), which regulate the immune function of the body and preserve immune homeostasis by maintaining the immune system's tolerance to itself, are well known. Among the four CAF subpopulations in the axillary lymph nodes of metastatic breast cancer patients mentioned previously, the CAF-S1 subpopulation was found to enhance the ability of Tregs to inhibit T-cell proliferation through B7H3, CD73 and DPP4, thus constituting an immunosuppressive environment in breast cancer cells [32]. In addition, in Treg-rich lung adenocarcinomas, where Tregs can produce high levels of TGF-β, CAFs also exhibit high TGF-β and VEGF expression. This in turn induces the apoptosis of CD8+T cells by inducing the conversion of NFs to CAFs, thereby promoting tumor escape from cytotoxic T-cells, and TGF-β can also promote the survival of Tregs [81]. In a recent study, IL1R2 was also found to promote TME immunosuppression during immune checkpoint inhibitor (ICI) treatment, and IL1R2 deficiency resulted in a decrease in the number of Tregs, an increase in the number of CD8+T cells, and a decrease in the number of exhausted CD8+T cells in the TME. IL1R2 is highly expressed on Tregs in the TME, while IL1R1 is mainly expressed on CAFs, which suggests that IL1R2 is involved in regulating the interaction between Tregs and CAFs and that IL1R2+Tregs in the TME promote the upregulation of MHC-II expression on CAFs [82]. MDSCs also exhibit potent immunosuppressive activity. In hepatocellular carcinoma, the cytokines IL-6 and SDF-1a secreted by CAFs can induce the production and activation of MDSCs, thus exerting an immunosuppressive effect, while IL-6 can upregulate the expression of PD-L1, thereby reducing its immunotherapeutic effect [83]. In colorectal cancer, CAFs with high FAP expression can also recruit MDSCs by upregulating CCL2 secretion through STAT3 signaling [84]. TAMs are the core of the immunosuppressive cell and cytokine network and play a crucial role in tumor immune evasion. TAMs can be divided into two subclasses, M1 and M2 [85]. M1 primarily mediates antibody-dependent cytotoxicity, while M2 has an immunosuppressive, procancer role in cell migration and invasion [86]. In breast cancer, CAFs release monocyte chemotactic protein 1 (MCP1), stromal cell-derived factor 1 (SDF-1) and chitinase-3-like protein 1 (CHI3L1) to promote the differentiation of monocytes into M2 macrophages. M2 macrophages can likewise secrete TGF-β to promote the transformation of endothelial cells to mesenchymal cells and to increase the responsiveness of CAFs, thus enhancing the invasiveness of cancer cells [87] (Fig. 2).

## CAFs and immunosuppressive cytokines

TGF-β is an important inhibitory cytokine within the TME, and its signaling pathways can be divided into two major categories: classical/Smad-dependent and nonclassical/Smad-independent pathways [88]. TGF-β primarily promotes tumor invasion and metastasis through EMT. TGF-β can be expressed by tumor cells and stromal cells, including CAFs, and it also activates CAFs, stimulates immunosuppression and promotes angiogenesis [7]. TGF-β enhances tumor invasion by enhancing MMP activity and promotes autocrine- and paracrine-mediated EMT induction [89]. According to the most recent research on pancreatic cancer, the combination of TGF-β inhibition and dual immune checkpoint inhibitors (ICIs) improved the immune response in pancreatic cancer patients [90]. TGF-β has also been found to stimulate angiogenesis by inducing the expression of VEGF-α [91]. In clinical trials, according to the finding that TGF-β suppresses T helper 2 (TH2)-cell-mediated cancer immunity, researchers have shown that blocking TGF-β signaling in CD4+T cells remodels the tumor microenvironment and restrains cancer progression [92]. CD8+T cells generated by IL-17α can promote PDAC by inducing the generation of inflammatory tumor-associated fibroblasts [93]. IL32 secreted by CAFs promotes the invasion and metastasis of breast cancer cells through integrin-3-p38 MAPK signaling [19]. In addition, inhibition of TGF-β signaling enhances responsiveness to immunosuppressive checkpoints and provides more possibilities for immunotherapy, which suggests that TGF-β is a highly desirable target [94]. Multiple cytokines, which are closely associated with CAFs, exhibit a wide range of antitumor activities [52, 92, 95]. In summary, in the TME, immune cells and immunosuppressive cytokines are inextricably linked with CAFs. We can utilize these findings to develop new treatment strategies and to improve the clinical application prospects of existing treatment methods. For example, specific CAF subsets can be targeted to inhibit the secretion of immunosuppressive cytokines. However, the signaling pathways in the tumor immune microenvironment are very complex and require further experimentation to develop better treatment strategies.

## Conclusion and future prospects

With an in-depth study of CAFs, we have gained a preliminary understanding of the various aspects of their heterogeneity and their immunosuppressive role in the TME, which will help in the development of immunotherapeutic strategies targeting CAFs as a new hope for cancer treatment. As more CAF subtypes are discovered, we should also pay attention to the nomenclature of different CAF subtypes and their proper characterization. The greatest potential of the future application of CAFs lies in targeting specific CAF subgroups. We can identify immunosuppressive or immunostimulatory CAF subtypes, such as CD105+CAFs, CD16+CAFs, and antigen-presenting CAFs through the use of specific biomarkers. The potential transition between immunosuppressive and immunostimulatory CAF subtypes has been further studied in the context of biomarkers or signaling pathways. Many clinical trials involving targeted treatment of CAFs alone or in combination with existing immunotherapies have been conducted. For example, some studies have shown that LRRC15^+^CAFs dependent on TGFβ dictate the tumor-fibroblast setpoint to promote tumor growth, and these cells also directly inhibit the function of CD8^+^ T-cells and limit the responsiveness to checkpoint blockade. As a potential therapeutic target, LCRRC15+CAFs can inhibit antitumor T-cell immunity and the effectiveness of ICB therapy [96]. However, despite substantial progress, many challenges remain, such as the lack of specific biomarkers for different subgroups of CAFs, the way in which the various immunosuppressive effects in the TME can be unlocked, and how to address the toxic side effects on other systems. In any case, the subpopulation characteristics of CAFs themselves need to be further understood, and more research is needed to determine the mechanisms that affect tumor immunosuppression and escape in the TME. Future exploration of specific subgroups of CAFs will lead to a new direction for targeted tumor therapy.

### Method

This review used PubMed to search the literature and Endnote X9 software for literature management.

## Data Availability

Not applicable.
